# Effects of nutritional intervention on insulin resistance-associated inflammatory biomarkers and gene polymorphisms in gestational diabetes mellitus

**DOI:** 10.5937/jomb0-63663

**Published:** 2026-05-22

**Authors:** Jiayu Li, Lili Sun, Linjing Zhang

**Affiliations:** 1 The First Affiliated Hospital of Hainan Medical University, Department of Obstetrics and Gynecology, Haikou City, Hainan Province, China; 2 Hainan Women and Children's Medical Center, Obstetrical Department, Haikou City, Hainan Province, China

**Keywords:** gestational diabetes mellitus, insulin resistance, inflammatory biomarkers, TNF-a, IL-6, adiponectin, nutritional intervention, gestacijski dijabetes melitus, insulinska rezistencija, inflamatorni biomarkeri, TNF-a, IL-6, adiponektin, nutritivna intervencija

## Abstract

**Background:**

Insulin resistance is a central biochemical abnormality in gestational diabetes mellitus (GDM) and is closely linked to chronic low-grade inflammation. Pro-inflammatory cytokines and adipokines, including tumor necrosis factor-a (TNF-a), interleukin-6 (IL-6), and adiponectin (APN), play key roles in metabolic dysregulation during pregnancy. This study investigated the effects of nutritional intervention on insulin resistance-related inflammatory biomarkers and their gene polymorphisms in pregnant women with GDM.

**Methods:**

In this retrospective comparative cohort study, 400 pregnant women diagnosed with GDM at Hainan Women and Children's Medical Center between October 2023 and December 2024 were enrolled and assigned to a conventional treatment group (CT, n = 200) or a nutritional intervention group (NC, n = 200). Both groups received standard glycemic management, while the NC group additionally underwent individualized nutritional intervention. Serum levels of TNF-a, IL-6, and APN were measured by enzyme-linked immunosorbent assay, and polymorphisms of the corresponding genes were analyzed using fluorescence quantitative PCR. Glycemic and lipid parameters were evaluated concurrently. Clinical efficacy and pregnancy outcomes were assessed as secondary endpoints.

## Abstract (sr)

**Uvod:**

Insulinska rezistencija je centralna biohemjska abnormalnost kod gestacijskog dijabetes melitusa (GDM) i usko j'e povezana sa hroničnom upalom niskog stepena. Proinflamatorni citokini i adipokini, uklj'učuj'ući faktor tumorske nekroze-a (TNF-a), interleukin-6 (IL-6) i adiponektin (APN), igraj'u klj'učnu ulogu u metaboličkoj' disregulaciji tokom trudnoće. Ova studija je istraživala efekte nutritivne intervencij'e na inflamatorne biomarkere povezane sa insulinskom rezistencij'om i nj'ihove genske polimorfizme kod trudnica sa GDM.

**Metode:**

U ovoj' retrospektivnoj' komparativnoj' kohortnoj' studiji, 400 trudnica sa dijagnozom gestacijskog dijabetesa (GD) u Medicinskom centru za žene i decu Haj'nan između oktobra 2023. i decembra 2024. godine uklj'učene su i raspoređene u konvencionalnu grupu za lečenje (KT, n = 200) ili grupu za nutritivnu intervencju (NK, n = 200). Obe grupe su primale standardno upravljanje glikemijom, dok je NK grupa dodatno podvrgnuta individualizovanoj nutritivnoj' intervencij'i. Serumski nivoi TNF-a, IL-6 i APN mereni su imunosorbentnim testom povezanim sa enzimima, a polimorfizmi odgovaraj'ućih gena analizirani su korišćenj'em fluorescentne kvantitativne PCR. Glikemij'ski i lipidni parametri su procenj'eni istovremeno. Klinička efikasnost i ishodi trudnoće procenjeni su kao sekundarni krajnji ciljevi.

**Rezultati:**

Distribucija genotipova TNF-a, IL-6 i APN nije pokazala značajne razlike između dve grupe (P &gt; 0,05). Početni inflamatorni, glikemijski i lipidni indeksi bili su uporedivi između grupa. Nakon intervencije, obe grupe su pokazale značajna poboljšanja inflamatornih markera, glukoze u krvi i lipidnih profila u poređenju sa početnim vrednostima (P &lt; 0,05). Primetno je da su nivoi TNF-a i IL-6 nakon tretmana bili značajno niži, dok su nivoi APN bili povoljnije regulisani u NC grupi u poređenju sa CT grupom (P &lt; 0,05). NC grupa je takođe pokazala superiornu metaboličku kontrolu i manju učestalost neželjenih ishoda trudnoće.

**Zaključak:**

Nutritivna intervencija značajno poboljšava inflamatorne i metaboličke profile povezane sa insulinskom rezistencijom kod trudnica sa gestacijskim dijabetesom (GDM), nezavisno od polimorfizama gena TNF-a, IL-6 i APN. Ovi nalazi ističu biohemijske koristi nutritivne modulacije u smanjenju upale i poboljšanju metaboličke homeostaze kod GDM.

## Introduction

Gestational diabetes mellitus (GDM), as the first detection or onset of diabetes during pregnancy, is of great concern because of its potential harm to both mother and foetus. Most patients lack obvious symptoms, and the abnormality is often detected by blood glucose testing at the time of delivery. Most of the abnormalities of glucose metabolism return to normal after delivery, but the risk of subsequent development of type 2 diabetes mellitus is significantly increased [1]. GDM not only threatens the health of the pregnant women themselves and is prone to problems such as hypertensive disorders in pregnancy, infections, and excessive amniotic fluid, but also increases the risk of adverse outcomes such as macrosomia, growth restriction, miscarriage, and preterm delivery for the fetus [2].

Insulin resistance plays a key role in the pathogenesis of GDM. Along with the advancement of pregnancy, insulin resistance in pregnant women is gradually aggravated [3]. In the early and middle stages of pregnancy, the foetal glucose uptake by the mother increases dramatically, while estrogen and progesterone levels rise significantly, leading to an increase in glucose utilisation by the mother, and the body's demand for glucose rises sharply. Moving into the middle and late stages of pregnancy, a variety of antagonistic insulin-like substances such as placental lactogen, oestrogen, progesterone, tumour necrosis factor and leptin are secreted in large quantities [4]. These substances interfere with the normal binding of insulin to cell surface receptors, impede the insulin signalling pathway, and dramatically reduce insulin sensitivity. Normally, pancreatic b-cells during pregnancy compensatorily increase insulin secretion to maintain blood glucose homeostasis. However, some pregnant women have poor pancreatic b-cell function, which makes it difficult for them to secrete enough insulin to counteract the increasing insulin resistance, leading to uncontrolled blood glucose and GDM [5]. Genetically, pregnant women with a family history of diabetes mellitus have a higher probability of carrying genes related to insulin resistance. Certain gene polymorphisms may affect the structure and function of insulin receptors or interfere with the expression of key proteins of insulin signalling, making them more likely to fall into the insulin resistance dilemma during pregnancy, which significantly increases the risk of developing GDM. In addition, factors such as obesity, polycystic ovary syndrome, poor maternal history, and advanced gestational age may also enhance the incidence of GDM by interfering with insulin sensitivity or secretion [6].

In terms of symptom presentation, GDM patients often present with symptoms of excessive drinking and frequent urination. Elevated blood glucose triggers osmotic diuresis, which leads to a large amount of water loss, and pregnant women have a strong sense of thirst, with a significant increase in the amount of water consumed and the frequency of urination [7]. Itchy skin is also common, as the hyperglycaemic environment facilitates bacterial growth in the skin tissues, leading to itching. In addition, patients are prone to fatigue, as insulin resistance prevents the conversion of sugar into energy, and the nutritional needs of the mother and foetus are extremely high during pregnancy, resulting in frequent fatigue and drowsiness [8]. However, most of the patients may not feel any discomfort, and the disease can only be detected with the help of blood glucose testing during labour and delivery [9]. The impact of GDM on the patient's daily activities is extensive and profound. In terms of diet, patients must strictly avoid foods high in sugar, fat, and salt, increase dietary fibre intake, and strictly enforce regular meals, which severely restricts the freedom of dietary choices and prevents them from enjoying their favourite food [10]. Exercise management has become a daily mandatory course, appropriate exercise helps to control blood glucose, but patients must carefully select the appropriate exercise mode and intensity according to their own conditions, such as walking, yoga for pregnant women, and also avoid exercise during fasting or high blood glucose. Frequent blood glucose monitoring also brings a lot of inconvenience, patients need to regularly measure fasting and postprandial blood glucose, and always pay attention to the fluctuation of blood glucose, which undoubtedly aggravates the psychological burden. Polydipsia and frequent urination seriously interfere with the quality of sleep, leading to mental depression, which greatly affects patients' energy for daily activities [11].

Currently, the treatment of GDM covers a variety of aspects such as drug therapy, insulin injection and lifestyle intervention [12]. In terms of pharmacological treatment, commonly used oral hypoglycaemic drugs such as sulfonylureas and biguanides can reduce blood glucose to a certain extent, but there are obvious drawbacks. For example, sulfonylureas are prone to drug failure with the increase in the number of years of the patient's illness, and if the diet and exercise are not matched properly, it is very easy to cause hypoglycaemia; biguanides may cause gastrointestinal discomfort, nausea, vomiting, abdominal pain and other symptoms, and long-term use of the drug (generally after 5 years) may also cause vitamin B12 deficiency [13]. For patients with type 2 diabetes mellitus who have severe complications of diabetes mellitus or for whom oral hypoglycaemic agents are ineffective, insulin injections are often required. However, insulin therapy has the problems of troublesome medication, poor experience, and high probability of hypoglycaemia, and obese patients may also aggravate the degree of obesity, while the improper injection technique is easy to cause the hardening of subcutaneous fat, which affects the absorption of insulin and reduces the effect of lowering glucose [14].

Although exercise therapy in lifestyle intervention is recommended for pregnant women to improve insulin sensitivity and control blood glucose through aerobic exercise such as walking and yoga for pregnant women, in practice, pregnant women often find it difficult to adhere to regular exercise due to physical inconvenience and fatigue during pregnancy. However, in practice, pregnant women often have difficulty in adhering to regular exercise due to physical inconvenience and pregnancy fatigue, and the effect of exercise on glycaemic control is limited, which makes it difficult to be used as an effective means of controlling GDM. In contrast, nutritional intervention has significant advantages in the treatment of GDM [15]. In terms of basic principles, it emphasises energy control, accurately determining daily energy intake based on the pre-pregnancy body mass index of pregnant women and the rate of growth of body mass during pregnancy, and adjusting it in due course through weekly monitoring of body weight. The pattern of regular meals at regular intervals helps to maintain relatively stable blood glucose throughout the day and effectively avoids the occurrence of hypoglycaemia. In terms of specific measures, preference is given to low glycaemic index foods, like wholemeal bread, brown rice and oats, which are slowly digested and absorbed, resulting in a smooth rise in blood glucose. Reasonable control of carbohydrate intake, reduce the intake of simple sugar foods such as candies and drinks, and increase the proportion of complex carbohydrates. At the same time, ensure adequate intake of high quality protein from sources such as lean meat, milk, eggs and beans to meet the needs of the pregnant woman herself and the foetus. Moderate intake of unsaturated healthy fats from olive oil, fish oil and nuts to reduce saturated fats and trans fats helps to maintain a good metabolic status [16]. In addition, more fresh vegetables and low-sugar fruits are consumed to ensure adequate intake of vitamins and dietary fibre. Through these scientific and reasonable dietary adjustments, nutritional intervention can not only effectively control blood glucose levels, but also provide a suitable nutritional environment for fetal growth and development, reduce the risk of maternal and infant complications, and help pregnant women maintain a reasonable weight gain without the side effects of medication and the inconvenience of insulin injections, which shows great potential for improving the condition and quality of life of GDM patients [17].

Currently, insulin resistance is a key factor in the pathogenesis of GDM, and there are certain limitations in the existing treatments, while nutritional intervention has significant advantages in improving the condition of GDM [18]. In view of the unique dietary culture and regional characteristics of Hainan Province, this study observed the genetic polymorphisms of inflammatory factors related to insulin resistance in pregnant women in Hainan Province, and analysed the efficacy of nutritional interventions in pregnant women in Hainan Province, which can provide a scientific basis for the prevention and treatment of GDM in this region.

## Materials and methods

### Study design

**Figure 1:**
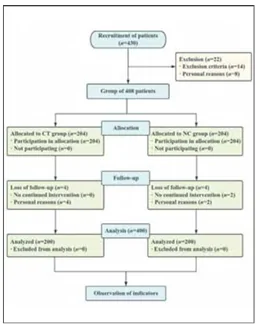
Flowchart of experimental design.

This is a systematic clinical retrospective study that observed the distribution of inflammatory factor genes associated with GDM in perinatal women, aiming to analyse the effect of nutritional intervention on GDM in perinatal women in Hainan Province. Four hundred pregnant women with GDM from Hainan Women and Children's Medical Center between October 2023 and December 2024 were selected and divided into two groups, the CT group and the NC group, according to different intervention modalities. Both groups were treated conventionally and nutritional intervention was added in NC group. A total of 430 patients' information was collected, 408 cases were included after exclusion, 8 cases were lost during the follow-up period, and finally a total of 400 cases were analysed. In this study, the therapeutic effect of nutritional intervention on GDM patients was analysed by comparison to provide a scientific basis for clinically relevant treatment protocols. The flow chart of this study is shown in Figure 1.

### Inclusion and exclusion criteria

Inclusion criteria: (1) singleton pregnancy; (2) Natural conception; (3) People at genetic risk for insulin resistance; (4) Age ≥25 years; (5) First-degree relatives with type 2 diabetes; (6) Overweight or obese (BMI ≥ 24 kg/m^2^); (7) History of GDM or history of delivery of a giant baby; (8) Patients with good overall mental status and basic health, who could truthfully express their complaints about symptoms and answer relevant questions from healthcare professionals; (9) The patient and his/her family are informed and agreeable, and sign an informed consent form.

Exclusion criteria: (1) Multiple pregnancies; (2) Artificial insemination; (3) Previous early pregnancy preservation; (4) Pre-pregnancy diabetes; (5) Hypertensive disorders during pregnancy; (6) Combination of serious heart, liver, kidney, lung and other important organ diseases; (7) Combination of major internal diseases such as systemic lupus erythematosus, thyroid disease, polycystic ovary syndrome, etc. that require long-term oral drug treatment; (8) Combined malignant tumour diseases; (9) Those with severe anaemia; (10) Those with mental illness, intellectual and speech disabilities; (11) Patients who have participated in clinical drug trials or clinical studies; (12) Those who request to stop treatment or are automatically discharged for personal reasons; (13) Other conditions that, in the opinion of the study physician, should not be included; (14) Other circumstances affecting the indicators of follow-up observation.

### Ethical statement

The study was performed in compliance with the Declaration of Helsinki and hospital ethical guideline and was endorsed by the hospital ethical committees.

### Interventions

Both groups were given standardised treatment, including regular antenatal checkups, participation in pregnancy-related health education and strict blood glucose monitoring. Healthcare professionals will regularly explain to pregnant women and their family members the basic knowledge of gestational diabetes, the potential hazards and the necessity of treatment, in order to reduce the psychological pressure of pregnant women. For pregnant women with excessive blood glucose levels, short-acting insulin injections (initially 4-6 U) are administered before meals, and the dosage is adjusted according to the results of consecutive blood glucose tests to ensure individualisation and effectiveness of treatment.

The NC group increased nutritional interventions with the following measures: (1) Nutritional assessment and dietary survey. At the initial visit of the pregnant women, the medical staff conducted a comprehensive nutritional assessment to understand the weight gain during pregnancy and their physical activity and obstetric examination results. In addition, pregnant women's exercise habits, dietary habits, meals in the last 3 d, nutritional status, nutritional knowledge and knowledge of blood glucose self-monitoring were assessed. Based on the assessment results, a personalised nutritional intervention plan was formulated. (2) Dietary structure adjustment and education. According to the daily energy demand of each pregnant woman, formulate a personalised diet plan and adjust the nutritional supply to ensure that the intake ratios of carbohydrates (200-300 g/d), proteins (80-100 g/d) and fats (50-70 g/d) are in line with the health guidelines. For pregnant women with gestational diabetes mellitus who are overweight or obese, the dietary structure of this group of pregnant women should be adjusted to increase the proportion of proteins and limit the intake of carbohydrates and fats in order to optimise the metabolic state and control weight gain. In addition, through one-on-one counselling, health education courses and distribution of educational brochures, the risk factors, potential threats and interventions of gestational diabetes mellitus should be explained to pregnant women in detail, stressing the importance of dietary and nutritional knowledge, and encouraging pregnant women to have regular follow-ups and actively cooperate with the treatment plan. (3) Dietary fibre and small and frequent meals. The intervention was conducted using a dietary fiber rich nutritional supplement independently developed by our research team. The preparation process of this supplement is as follows: ① Raw material pretreatment: Select oat **b**-glucan, resistant dextrin, and oligofructose with a purity of ≥ 95% as the core raw materials, and dry them at 60°C for 2 h to remove free water from the raw materials; ② Accurate ratio: Weigh oat **b**-glucan, resistant dextrin, and oligofructose in a mass ratio of 14:37:49, mix evenly, and sieve through an 80 mesh sieve to ensure uniform particle size; ③ Formulation molding: The mixed powder is processed into 15 g/part granules using dry granulation technology, and stored in a sealed manner away from light. Each serving is 15 g and contains approximately 46.5 kcal of energy, 1.7 g of protein, 0.3 g of fat, 3.7 g of carbohydrates, 9.5 g of dietary fiber, 8.6 mg of sodium, and 1.4 g of oat beta glucan. Take with 150 mL of drinking water, 1 or 2 times/d. In addition, healthcare professionals will advise pregnant women to distribute meals in the form of 3 main meals and 2 to 3 additional meals per day to help maintain stable blood glucose levels, and to give preference to low-sugar coarse grains, such as buckwheat and oats, as well as fibre-rich fresh vegetables and low glycemic index fruits whenever possible to optimise postprandial glycaemic response. ④ Monitoring and adjustment. Intensive monitoring of blood glucose levels in pregnant women and adjusting the dietary regimen according to blood glucose fluctuations. Review every 2 weeks and evaluate the blood glucose and dietary situation and the implementation rate of the nutritional intervention programme at the next review, provide feedback to the pregnant women on the results and make timely adjustments. After 24 weeks of pregnancy, according to the pregnant woman's condition, a daily calcium supplement of 600 mg is recommended. This dose refers to the recommended calcium intake standard for women in the middle and late stages of pregnancy in the »Reference Intake of Dietary Nutrients for Chinese Residents« (the recommended calcium intake for women in the middle and late stages of pregnancy is 1000 mg/d), combined with the baseline level of calcium intake in the daily diet of the study subjects (about 400 mg/d), to ensure that the total daily calcium intake meets the standard [19]. Remind pregnant women to keep a good record of their diet, and avoid high-fat and high calorie foods as much as possible. They should also supplement foods rich in trace elements, vitamin B, and folic acid appropriately. On average, a nutritional intervention programme should be implemented for ≥3 d in 1 week.

The intervention was continued until delivery in both groups.

### Inflammatory factor gene information

### DNA extraction

DNA extraction of patient serum samples was performed using the silica gel plate method [20]. Centrifuge tube barcode clarity and integrity were first verified, followed by 12 h overnight lysis of the samples. The 96-well collection plate was placed in a deep-well waste plate, and the centrifuge tube solution was transferred sequentially. After extraction, the DNA samples were sealed in a 96-well shallow-well plate, and the self-adhesive sheets were glued and handed over to the production personnel. Simultaneously record the sample number, check the number of samples, volume and batch number, scan the barcode and register it in the 'DNA Product Preservation Sheet', print it and hand it over to the custodian for filing together with the nucleic acid sample plate.

### Fluorescence quantitative PCR assay

### (1) Reaction system construction

After the 10X probe plate, Master mix and DNA samples were melted at room temperature, shaking and mixing for 10 s, centrifuge at 900 rpm for a few moments. The reaction system was prepared according to the sample volume (total volume 6 mL: Master mix 3.1 mL, probe 0.16 mL, ddH_2_O 1.94 mL, DNA 1.0 mL). The reagents were added sequentially to a sterile 96-well plate (cut to 48 wells), sealed, vortexed for 5 s, and centrifuged at 4000 rpm for 1 min to ensure that the liquid settles to the bottom. The reaction system was dispensed into 384-well plates, and the DNA samples were added and centrifuged again, and then placed in a preheated 9700 PCR instrument and amplified according to the set procedure (95°C for 10 min pre-denaturation; 92°C for 15 s, 60°C for 1 min, 20 cycles; 89°C for 15 s, 60°C for 1 m for 30 s, 30 cycles; stored at 4°C).

The reagents were selected from Taqman fluorescent probes (APN, IL-6, TNF-a) of ABI, USA, to ensure the standardisation and reliability of the detection system.

### (2) Endpoint plate reading and data analysis

Using ABI 7900 fluorescence quantitative PCR instrument (ABI), after preheating, connecting the equipment, loading the 384-well plate to read the plate operation, the results are named 'production batch number + probe number' to save and burn the disc. Import the barcode and probe information in the special computer for analysis, tick the sample probe according to the spiking distribution table, set the negative control (NTC), mark the sample genotype after analysis by the software, export the data and save the file.

### (3) Quality control

Daily take the quality control product room temperature equilibrium 15 min with the sample detection, if there is a loss of control of quality control, immediately start the deviation processing procedures, withholding the test results, to be troubleshooting after re-measurement. Monthly summary of quality control data to form a report, and strict verification of reagent expiration date, to prevent the use of expired reagents.

### Observational indicators

### Inflammation indicators

Before and after treatment, 3 mL of fasting peripheral venous blood was drawn from the patients, centrifuged and processed, and then kept refrigerated to be tested, and enzyme-linked immunosorbent assay (ELISA) was used to determine and calculate the levels of tumour necrosis factor-a (TNF-a), interleukin-6 (IL-6), and adiponectin (APN) in the patient's serum samples [21].

The kits used were Human TNF-a ELISA kit (Item No.: ml077385, Shanghai Enzyme-linked Biotechnology Co., Ltd., Shanghai, China), Human IL-6 ELISA kit (Item No.: ml027379, Shanghai Enzyme-linked Biotechnology Co., Ltd., Shanghai, China) and Human APN ELISA kit (Item No.: ml105581, Shanghai Enzyme-linked Biotechnology Co., Ltd., Shanghai, China). According to the manufacturer's instructions, the intra-assay coefficients of variation (CVs) of the three kits were ≤ 8.0%, and the inter-assay CVs were < 10.0%, which indicated good repeatability and reliability of the detection results.

### Blood glucose indicators

About 5 mL of fasting venous blood was collected from all patients respectively, and the serum was taken after waiting for centrifugation. Blood glucose meter (GA-8, Sanno Biosensing Co., Ltd, device registration number: 20162400156) and glycated haemoglobin analyser (H-120P Myriad, device approval number: 20252220009) were used to measure 2 h postprandial blood glucose (2 h PG), glycated haemoglobin (HbA1c), and fasting blood glucose (FPG) [22][23].

### Lipid indicators

Fasting venous blood was collected from all patients, and the levels of triacylglycerol (TG), total cholesterol (TC), and low-density lipoprotein cholesterol (LDL-C) were measured by a fully automated biochemical analyser (BS-220, Myriad, National Instrument Registration No.: 20192220492) [24].

### Clinical efficacy

Compare the clinical efficacy of the two groups. Efficacy assessment criteria: Obvious effect: TNF-a and IL-6 levels decreased by ≥40%, APN levels increased by ≥30%, blood glucose indexes decreased significantly, lipid levels improved by ≥20%, and patients' symptoms related to inflammation, hyperglycemia, and hyperlipidemia basically disappeared. Effective: TNF-a and IL-6 levels decreased by 20%~40%, APN levels increased by 15%~30%, blood glucose indicators decreased, blood lipid levels improved by 10%~20%, and patients' related symptoms reduced. Ineffective: the changes of the above indexes did not reach the effective standard, or TNF-a, IL-6, blood glucose level did not decrease or even increased, blood lipid level did not improve, APN, patient's symptoms did not improve or even worsened. Overall effective rate = (obvious effect + effective) / total X 100%.

### Quality of life

The quality of life of patients in both groups was compared using the Short Form of the Health Status Survey (SF-36 score) in the dimensions of physiological functioning, physical functioning, somatic pain, general health status, social functioning, emotional functioning, and mental health, with a total score of 100 points, with higher scores indicating a better quality of life [25].

### Nursing care satisfaction

To compare the two groups of patients' satisfaction with postoperative intervention care, a homemade nursing satisfaction questionnaire was used to assess the four dimensions of professionalism, nursing operation, communication and attitude, with a total score of 25 points for each dimension, with higher scores indicating higher satisfaction with nursing care in that dimension.

### Adverse pregnancy outcomes

Adverse pregnancy outcomes were recorded in both groups after the intervention. Including gestational hypertension, ketoacidosis, excessive amniotic fluid, macrosomia, low-birth-weight neonates, intrauterine fetal death, neonatal distress, neonatal hypoglycemia, preterm birth, fetal malformations and so on.

Among them, the diagnosis of fetal abnormalities requires systematic ultrasound screening at 20-24 weeks of pregnancy (according to the ISUOG standard) [26], combined with fetal genetic testing if necessary, and comprehensive judgment of post-partum physical examination and auxiliary examinations.

### Follow-up visits

In this study, follow-up visits were arranged mainly at 3 months after treatment to assess the durability of the effect and to address any potential adverse reactions or problems.

### Sample size calculation

The sample size was based on a power analysis using G*Power 3.1.9.7 computer software to determine the sample size required to detect a statistically significant difference. The sample size was calculated based on the primary outcome of clinical efficacy. Considering an alpha level of 0.05 and 90% efficacy, we calculated that a sample size of 136 patients was required for each group. Considering the potential uncertainties, the sample sizes selected for this study were the CT group (*n* = 200) and the NC group (*n* = 200), and we believe that the sample sizes in this study allow for reliable conclusions to be drawn.

### Statistical analysis

Statistic Package for Social Science (SPSS) 28.0 statistical software (IBM, Armonk, NY, USA) was used for data analysis. Lucidchart was used to draw flowcharts. The data in this study were tested for normal distribution. Baseline characteristics were described as number of people and variables (expressed as x̄±s). The results of inflammatory indicators, glycaemic indicators, lipid indicators and SF-36 scores results in the results are expressed as x̄±s. Comparison between the two groups was examined using independent samples *t*-test. The clinical efficacy, the incidence of complications and adverse reactions in the results were expressed as proportions (%). Comparison among both groups was analysed using *x^2^* test. All statistical tests were two-sided, and *P*<0.05 indicated a statistically significant difference.

## Results

### Basic information

In this study, 400 pregnant women with GDM from Hainan Women and Children's Medical Center between October 2023 and December 2024 were assigned to CT group (*n* = 200) and NC group (*n*=200) according to different interventions, aiming to investigate the differences in the efficacy of different interventions for pregnant women with GDM. The baseline demographic and baseline characteristics of the patients in both groups are presented in Table 1, which were statistically analysed using independent samples t-test (for continuous variables) and chi-square test (for categorical variables), and the results showed that the baseline characteristics of each group did not differ markedly between the both groups (*P*>0.05). This indicates that the two groups were well comparable before treatment, and the confounding effects of demographic and clinical factors were effectively controlled in this study, with minimal impact on the analysis of the results, thus guaranteeing the reliability and accuracy of the subsequent efficacy evaluation.

**Table 1:** Patient demographics and baseline disease characteristics.

Parameter		CT group (*n*=200)	NC group (*n*=200)	*t/x^2^*	*P*
Age (year)		33.24±4.72	32.85±5.21	-0.785	0.433
Height (year)		158.94±3.87	158.86±3.71	-0.211	0.833
Weight (kg)		65.76±3.99	65.25±3.85	-1.301	0.194
Pre-pregnancy BMI (kg/m^2^)		21.68±2.11	21.99±1.77	1.592	0.112
Early pregnancy (Yes/no)		91/109	102/98	0.599	0.439
Birth ≥1 (Yes/no)		110/90	114/86	0.081	0.776
Multiple births (Yes/no)		3/197	4/196	0.000	0.996
History of miscarriage (Yes/no)		42/158	41/159	0.000	0.986
Three months <br>prior to <br>pregnancy	Passive smoking (Yes/no)	46/154	48/152	0.024	0.877
	Alcohol consumption (Yes/no)	6/194	5/195	0.000	0.995
Family <br>history	GDM (Yes/no)	115/85	105/95	0.500	0.480
	Hypertension (Yes/no)	32/168	35/165	0.118	0.731
	Diabetes (Yes/no)	16/184	20/180	0.233	0.630
Occupation (Manual labour/mental labour)		85/115	89/111	0.081	0.777
Ethnicity (Han Chinese/Minority)		189/11	196/4	2.042	0.153
Payment method (With/without medical insurance)		158/42	159/41	0.001	0.971
Temperature (°C)		36.46±0.45	36.49±0.46	0.659	0.510
Respiration (breaths/min)		17.55±1.51	17.46±1.44	-0.610	0.542
Heart rate (beat/min)		75.06±5.06	74.86±4.88	-0.402	0.688
Systolic blood pressure (mmHg)		119.47±4.88	119.65±5.17	0.358	0.721
Diastolic blood pressure (mmHg)		75.84±4.30	75.41±4.14	-1.019	0.309

### Inflammatory factor gene distribution

The base distribution of the TNF-a gene locus includes three genotypes: AA, AG and GG. The base distribution of IL-6 gene locus covers three genotypes: GC, CC and GG. The base distribution of APN gene locus includes three genotypes: GT, TT and GG. The genotypes of TNF-a, IL-6 and APN loci in the two groups were statistically analysed, and the results were presented in Table 2. The genotypes of AA, AG, and GG for TNF-a, GC, CC, and GG for IL-6, and GT, TT, and GG for APN were not markedly different among both groups (P > 0.05). This indicates that the genotype distribution of the above inflammatory factor genes did not differ remarkably among both groups within the sample of this study, suggesting that the polymorphisms of these genes may not have a significant effect on the regulation of inflammatory factor expression and subsequent biological effects in the relevant diseases or states of interest in this study.

**Table 2:** Basic distribution (%).

Norm	Nucleobase	CT group	NC group	x^2^	*P*
TNF-a	AA	0.5	1	0.000	0.997
	AG	19	17.5	0.036	0.851
	GG	80.5	81.5	0.032	0.859
IL-6	GC	28.5	35	0.915	0.339
	CC	64.5	56	1.464	0.226
	GG	7	9	0.272	0.602
APN	GT	46	44	0.081	0.776
	TT	45.5	44	0.025	0.875
	GG	8.5	12	0.512	0.474

### Inflammation indicators

The results of the comparison of the inflammatory indicators of both groups are presented in Table 3. Before treatment, no remarkable discrepancy was found in the inflammatory index levels of both groups when compared among the groups (*P*>0.05). Post-treatment, the levels of inflammatory indicators in the CT group were (15.60±3.10) mg/L, (18.64±5.03) mg/L and (18.02±0.93) mg/mL, and in the (10.14±3.08) mg/L, (9.60±4.49) mg/L and (13.07±1.15) mg/L in the NC group, respectively, were all markedly reduced from pre-treatment, and the NC group was markedly below the CT group (*P*<0.05). It indicated that the inflammatory indicators levels of both groups were decreased remarkably after treatment, and the improvement was better in NC group patients.

**Table 3:** Comparisons of inflammation indicators (x̄±s). Note: »*« represents marked discrepancy compared with pre-treatment, P<0.05.

Norm	Time	CT group	NC group	*t*	*P*
TNF-a (mg/L)	Pre-treatment	27.97±3.85	28.03±3.84	0.156	0.876
	Post-treatment	15.60±3.10*	10.14±3.08*	-17.670	<0.001
IL-6 (mg/L)	Pre-treatment	34.01±5.52	34.53±4.96	0.991	0.322
	Post-treatment	18.64±5.03*	9.60±4.49*	-18.961	<0.001
APN (mg/L )	Pre-treatment	28.02±1.56	27.99±1.40	-0.202	0.840
	Post-treatment	18.02±0.93*	13.07±1.15*	-47.332	<0.001

### Blood glucose indicators

The results of the comparison of the blood glucose indicators of both groups of patients are presented in Table 4. Before treatment, no remarkable difference was found in the blood glucose index levels of both groups compared with each other (*P*>0.05). Post-treatment, the blood glucose index levels of CT group were (7.71±1.44) mmol/L, (6.43±1.22)% and (5.65±0.90) mmol/L, and (6.00±1.05) mmol/L, (5.01 ±1.03)% and (3.99±0.83) mmol/L in the NC group, respectively, were all markedly reduced compared with pre-treatment, and the NC group was markedly below the CT group (*P*<0.05). This indicates that the levels of glycaemic indexes of patients in both groups were decreased remarkably after treatment, and the improvement of patients in NC group was better.

**Table 4:** Comparisons of blood glucose indicators (x̄±s). Note: »*« represents marked discrepancy compared with pre-treatment, P<0.05.

Norm	Time	CT group	NC group	*t*	*P*
2 h PG (mmol/L)	Pre-treatment	9.92±1.57	9.89±1.54	-0.193	0.847
	Post-treatment	7.71±1.44*	6.00±1.05*	-13.570	<0.001
HbA1c(%)	Pre-treatment	7.98±1.46	7.96±1.32	-0.144	0.886
	Post-treatment	6.43±1.22*	5.01±1.03*	-12.577	<0.001
FPG (mmol/L )	Pre-treatment	8.71±1.35	8.54±1.42	-1.227	0.221
	Post-treatment	5.65±0.90*	3.99±0.83*	-19.175	<0.001

### Blood lipid index

The results of the comparison of the blood lipid indexes of both groups of patients are presented in Table 5. Pre-treatment, no remarkable difference was found in the levels of lipid indexes of both groups (*P*>0.05). Post-treatment, the lipid index levels of CT group were (1.99±0.65) mmol/L, (4.66±0.58) mmol/L and (3.06±0.61) mmol/L, and (1.70±0.53) mmol/L, (4.17±0.54) mmol/L and (2.54±0.62) mmol/L in the NC group, respectively, were all remarkably decreased from pre-treatment, and the NC group was remarkably below the CT group (*P*<0.05). It indicated that the levels of lipid indexes were decreased remarkably in both groups after treatment, and the improvement of lipid indexes was better in NC group patients.

**Table 5:** Comparisons of blood lipid index (x̄±s, mmol/L). Note: "*" represents marked discrepancy compared with pre-treatment, P<0.05.

Norm	Time	CT group	NC group	*t*	*P*
TG	Pre-treatment	2.50±0.95	2.55±0.99	0.515	0.607
	Post-treatment	1.99±0.65*	1.70±0.53*	-4.890	<0.001
TC	Pre-treatment	5.23±1.12	5.36±1.26	1.091	0.276
	Post-treatment	4.66±0.58*	4.17±0.54*	-8.744	<0.001
LDL-C	Pre-treatment	3.33±0.58	3.41±0.62	1.333	0.183
	Post-treatment	3.06±0.61*	2.54±0.62*	-8.455	<0.001

### Clinical efficacy

Combined with the therapeutic effect we analysed the clinical efficacy of the two groups of patients, the results of the analysis are shown in Table 6. The total effective rate of the NC group patients was 92.00% (184/200), and the CT group was 80.00% (160/200). The comparison between the groups had a remarkable difference (*P*<0.05). The results showed that the NC group patients had better efficacy, indicating that the clinical efficacy of the combined treatment was better.

**Table 6:** Clinical efficacy analysis.

Group	Obvious effect <br>(*n*)	Effective<br>(*n*)	Ineffective<br>(*n*)	Total efficacy rate <br>(*n, %*)
CT group	70	90	40	160 (80.00)
NC group	84	100	16	184 (92.00)
*x^2^*	6.240			
*P*	<0.05			

### Quality of life

The results of the comparison of the quality of life scores of both groups are presented in Table 7. Before treatment, no remarkable difference was found in the SF-36 scores of patients in both groups (*P*>0.05). After treatment, the SF-36 scores of patients in both groups were improved, and the SF-36 score of the NC group was (94.76±3.30), which was remarkably greater than the CT group's (89.84±3.63), with a remarkable difference (*P*<0.001). It indicates that the combination therapy in this study can effectively improve the quality of life of patients.

**Table 7:** Comparisons of SF-36 scores (x̄±s, score).

	CT group	NC group	*t*	*P*
Pre-treatment	82.88±2.93	83.27±3.08	1.297	0.195
Post-treatment	89.84±3.63	94.76±3.30	14.183	<0.001
*t*	21.100	35.997		
*P*	<0.001	<0.001		

### Nursing satisfaction

Comparing the nursing satisfaction of both groups of patients with the intervention treatment of this study, the results of the questionnaire are presented in Table 8, and there is a marked difference in the satisfaction of both groups in four aspects, including the professionalism of the nursing staff and the nursing operation, and the patients of the NC group have a higher score of satisfaction with all nursing than the CT group (*P*<0.05), which suggests that the patients of the NC group are very satisfied with the programme of the nutritional intervention treatment.

**Table 8:** Nursing care satisfaction (x̄±s, score).

	Professionalism	Nursing practice	Communication	Attitude
CT group	18.81±2.06	18.90±1.98	18.83±2.02	19.40±1.80
NC group	21.19±1.92	22.46±1.46	21.90±1.96	22.49±1.74
*t*	11.952	20.465	15.425	17.455
*P*	<0.001	<0.001	<0.001	<0.001

### Adverse pregnancy reactions

We followed up the patients to observe the adverse reactions. The occurrence of adverse pregnancy reactions during treatment in both groups is presented in Table 9. No remarkable difference was found in the comparison of adverse reactions such as pregnancy hypertension in patients of both groups (*P*>0.05). The total incidence of patients in the CT group who developed adverse reactions was 15.00% (30/200), which was remarkably greater the 5.00% (10/200) of patients in the NC group (*P*<0.05), indicating that the therapeutic efficacy of the treatments used in patients in the NC group was better and safer.

**Table 9:** Adverse pregnancy reactions (n(%)).

	CT group	NC group	*x^2^*	*P*
Hypertension in pregnancy	3 (1.50)	1 (0.50)	0.338	0.561
Ketoacidosis	2 (1.00)	1 (0.50)	0.000	0.994
Excessive amniotic fluid	3 (1.50)	1 (0.50)	0.338	0.561
Macrosomia	4 (2.00)	2 (1.00)	0.338	0.561
Low birth weight neonate	3 (1.50)	0 (0.00)	2.000	0.157
Intrauterine foetal death	4 (2.00)	1 (0.50)	0.349	0.555
Neonatal distress	2 (1.00)	1 (0.50)	0.000	0.994
Neonatal hypoglycaemia	3 (1.50)	1 (0.50)	0.338	0.561
Preterm labour	3 (1.50)	1 (0.50)	0.338	0.561
Fetal anomalies	3 (1.50)	1 (0.50)	0.338	0.561
Overall incidence	30 (15.00)	10 (5.00)	5.556	<0.05

## Discussion

GDM is a serious threat to the health of mothers and infants, and insulin resistance occupies a central role in the development of GDM. Hormone levels change dramatically in women during pregnancy and they reduce the sensitivity of maternal tissues to insulin to varying degrees, making insulin much less efficient in promoting glucose uptake and utilisation [27]. This insulin resistance worsens as the weeks of pregnancy increase, and when insulin secretion from the pancreas fails to increase compensatingly to overcome the resistance, blood glucose cannot be metabolised properly, which in turn triggers GDM [28]. In terms of clinical features, pregnant women with GDM who have insulin resistance tend to have normal or only mildly elevated fasting blood glucose, but significantly elevated postprandial blood glucose, presenting a glucose profile dominated by postprandial hyperglycaemia. Studies have shown that such pregnant women have a significantly increased risk of macrosomia, fetal growth restriction, preterm delivery, miscarriage, hypertensive disorders of pregnancy, and maternal and fetal complications such as neonatal respiratory distress syndrome and neonatal hypoglycaemia, as compared to pregnant women with normal insulin sensitivity [29]. Genetic factors are also strongly associated with insulin resistance in GDM patients. Certain genetic polymorphisms may affect the expression or function of key proteins in the insulin signalling pathway, altering the body's responsiveness to insulin and increasing the chance of insulin resistance. In addition, environmental factors such as over-nutrition, insufficient exercise and obesity during pregnancy can further aggravate insulin resistance and contribute to the development of GDM [30].

In view of the close connection between insulin resistance and GDM, clinical prevention and treatment for GDM are also mostly centred on improving insulin resistance [31]. In terms of diet, emphasis is placed on the rational allocation of the proportion of carbohydrates, proteins and fats, and increasing dietary fibre intake to stabilise blood glucose levels; in terms of exercise, pregnant women are encouraged to engage in appropriate aerobic exercise, such as walking and yoga for pregnant women, to enhance the body's sensitivity to insulin; and, if necessary, medications, such as insulin, will be used to control blood glucose and to improve the state of insulin resistance, reducing the occurrence of adverse outcomes for both mothers and infants. Insulin resistance is the core link in the pathogenesis of GDM, and in-depth investigation of its mechanism is of great significance in enhancing the early diagnosis, precise treatment and improving the prognosis of mothers and infants in GDM, and also provides an important direction for the optimisation of related prevention and treatment strategies in the future [32].

TNF-a, IL-6 and APN gene polymorphisms are closely associated with insulin resistance and inflammatory response, and previous studies have shown that specific genotypes of these genes may influence the risk of GDM [33]. In the current study, analyses for the AA, AG, and GG genotypes of the TNF-a gene, the GC, CC, and GG genotypes of the IL-6 gene, and the GT, TT, and GG genotypes of the APN gene in the two groups of patients were analysed, and the results showed that there was no significant difference (P>0.05). This result is inconsistent with the findings of some studies and may be related to the unique genetic background and living environment of the population in the Hainan region. The dietary structure, lifestyle and environmental factors in the Hainan region may have a modifying effect on gene expression, weakening the impact of genotypic differences on disease. In addition, factors such as sample size and the sensitivity of genetic testing methods may also interfere with the results. The results suggest that polymorphisms in the TNF-a, IL-6 and APN genes may not be the key factors influencing the development of GDM and insulin resistance in pregnant women in Hainan, and that subsequent studies could expand the sample size and incorporate more environmental factors in order to further explore their associations with the effects of nutritional interventions.

GDM, as a common disorder of abnormal glucose metabolism during pregnancy, has many potential risks to the health of mothers and infants. This study focuses on the effects of nutritional interventions on insulin resistance and related inflammatory factors in pregnant women in Hainan Province, in which changes in inflammation, blood glucose and lipid indicators are significant. Inflammatory indicators play an important role in the pathogenesis of GDM. Inflammatory state can exacerbate insulin resistance and interfere with normal glucose metabolism [34]. Pre-treatment inflammatory indexes of both groups had no remarkable difference, indicating that both groups were well comparable at the beginning of the study. Post-treatment, the inflammatory indexes of both groups were remarkably reduced, indicating that the treatment was effective in controlling inflammation, while the NC group was reduced more significantly, showing the unique advantage of nutritional intervention in suppressing inflammation. This may be due to the rational nutritional allocation, optimizing the body's immune and metabolic microenvironment, which in turn effectively suppressed the inflammatory response, providing favourable conditions for improving insulin sensitivity and regulating glucose metabolism. Yu et al. [35] reported in a meta-analysis study on the effects of different nutrients on blood glucose, inflammatory reaction and oxidative stress in pregnant diabetes that inflammatory indicators were significantly reduced after nutritional intervention, which was similar to the findings of the present study.

Blood glucose index is the key to measure the condition of GDM and treatment effect. The blood glucose levels of both groups were comparable before treatment, and both groups decreased remarkably after treatment, and the NC group had better blood glucose control. Effective control of blood glucose is essential to reduce the risk of complications in pregnant women with GDM, such as reducing the occurrence of adverse outcomes such as macrosomia, neonatal hypoglycaemia and fetal distress [36]. Nutritional interventions help pregnant women maintain stable blood glucose and reduce the adverse effects of blood glucose fluctuations on the health of mother and child through scientific planning of dietary structure and calorie intake. Dyslipidaemia is closely related to GDM, and increased estrogen levels and enhanced intestinal absorption of lipids during pregnancy often lead to elevated blood lipids [37]. In this study, there was no difference in lipid indexes in both groups pre-treatment, and both groups were reduced after treatment, with a more pronounced reduction in the NC group. Effective control of blood lipids can reduce the risk of hypertension, cardiovascular disease and acute pancreatitis during pregnancy, and also reduce the occurrence of macrosomia and preterm labour. Nutritional interventions play a role in limiting salt, limiting sugar and adjusting the proportion of fatty acid intake to improve the lipid profile.

In addition, the NC group was better than the CT group in terms of total effective rate of treatment, SF - 36 score, nursing care satisfaction, and the incidence of adverse reactions was lower, which fully demonstrated that the nutritional intervention not only can effectively improve the clinical indicators of pregnant women with GDM, but also can enhance their quality of life and nursing care experience, and it is highly safe. The results show that nutritional intervention has an important clinical value in improving the health status of women during peri-pregnancy, and it is worth promoting and applying in clinical practice to better protect the health of mothers and infants. From the perspective of promotion feasibility, this intervention plan is easy to operate, cost-effective, and suitable for primary healthcare resources. To address the issue of insufficient nutrition professionals at the grassroots level, standardized training can be provided to enhance the intervention capabilities of medical staff. At the same time, requirements such as »small meals, multiple meals« and »dietary records« are easy to implement. Combined with regular follow-up visits and blood glucose monitoring feedback, patient compliance can be improved. In the future, the implementation of the plan can be further promoted through community health management models.

This study has certain limitations. First, this study is a single center retrospective study with a relatively limited sample size. It is not only difficult to completely cover the different severity grades and course stages of insulin resistance and gestational diabetes (GDM) in pregnant women, but also fails to fully include patients with multiple system complications and stratified subjects of different gestational weeks, which may introduce selective bias, and will also weaken the extrapolation applicability of the research conclusions and reliability in a variety of clinical scenarios. Second, although there was no significant difference in the baseline data between the groups before treatment, there was still individual heterogeneity in patients' lifestyle habits, genetic background, and underlying diseases, and these uncontrolled confounding factors may interfere with the assessment of the real effects of nutritional interventions and affect the generalisability of the study results. In addition, the follow-up period of the study only focused on the short-term changes in indicators after treatment, and failed to track the metabolic status of the patients in the months or even years after delivery, as well as the health of the offspring and other long-term outcomes, which did not allow for an adequate assessment of the long-term effectiveness and safety of nutritional interventions. Based on this, it is advisable to further expand the sample size to include pregnant women with different characteristics and adopt a multi-centre study design to enhance the representativeness of the study; at the same time, it is also advisable to extend the follow-up period, establish a long-term health tracking mechanism, and combine the analysis of gene expression dynamics with the monitoring of lifestyle, so as to more comprehensively and accurately assess the value of nutritional interventions in preventing and treating GDM in pregnant women in Hainan Province.

## Conclusion

In summary, this study shows that in pregnant women in Hainan Province, nutritional intervention can more significantly reduce the levels of inflammation, blood glucose and lipid indicators, enhance the overall effective rate of treatment, improve patients' quality of life and satisfaction with care, and reduce the incidence of adverse reactions, which has a positive effect on improving insulin resistance and related inflammatory states. Meanwhile, the distribution of TNF-a, IL-6 and APN gene polymorphisms in both groups was not significantly different, suggesting that they may not be the key factors influencing the occurrence of insulin resistance and GDM in pregnant women in this region. This study provides a practical basis for nutritional intervention for the prevention and treatment of GDM in pregnant women in Hainan, but the relationship between gene polymorphisms and the effect of nutritional intervention still needs further in-depth study. However, the present study has a small sample size and a short follow-up period, which fails to observe the long-term effectiveness of this method of treatment. Multi-centre, large-sample, high-quality clinical studies can be continued for validation in the later stage.

## Dodatak

### Acknowledgment

None.

### Consent to publish

The manuscript has neither been previously published nor is under consideration by any other journal. The authors have all approved the content of the paper.

### Consent to participate

We secured a signed informed consent form from every participant.

### Ethic approval

This study was approved by the Ethics Committee of the Hainan Women and Children's Medical Center.

### Data availability statement

The data that support the findings of this study are available from the corresponding author, upon reasonable request.

### Funding

None.

### Conflict of interest statement

All the authors declare that they have no conflict of interest in this work.

### Contribution

Jiayu Li and Lili Sun contributed equally to this work.
